# Flame Retardants-Mediated Interferon Signaling in the Pathogenesis of Nonalcoholic Fatty Liver Disease

**DOI:** 10.3390/ijms22084282

**Published:** 2021-04-20

**Authors:** Chander K. Negi, Sabbir Khan, Hubert Dirven, Lola Bajard, Luděk Bláha

**Affiliations:** 1 Faculty of Science, RECETOX, Masaryk University, Kamenice 5, CZ62500 Brno, Czech Republic; lola.bajard@recetox.muni.cz (L.B.); ludek.blaha@recetox.muni.cz (L.B.); 2 Department of Neuro-Oncology, The University of Texas MD Anderson Cancer Center, 1515 Holcombe Boulevard, Houston, TX 77030, USA; ksabbir25@gmail.com; 3 Department of Environmental Health, Section for Toxicology and Risk Assessment, Norwegian Institute of Public Health, 0456 Oslo, Norway; hubert.Dirven@fhi.no

**Keywords:** flame retardants, nonalcoholic fatty liver disease, metabolism-disrupting chemicals, cytokines, interferon, metabolic disruption

## Abstract

Nonalcoholic fatty liver disease (NAFLD) is a growing concern worldwide, affecting 25% of the global population. NAFLD is a multifactorial disease with a broad spectrum of pathology includes steatosis, which gradually progresses to a more severe condition such as nonalcoholic steatohepatitis (NASH), fibrosis, cirrhosis, and eventually leads to hepatic cancer. Several risk factors, including exposure to environmental toxicants, are involved in the development and progression of NAFLD. Environmental factors may promote the development and progression of NAFLD by various biological alterations, including mitochondrial dysfunction, reactive oxygen species production, nuclear receptors dysregulation, and interference in inflammatory and immune-mediated signaling. Moreover, environmental contaminants can influence immune responses by impairing the immune system’s components and, ultimately, disease susceptibility. Flame retardants (FRs) are anthropogenic chemicals or mixtures that are being used to inhibit or delay the spread of fire. FRs have been employed in several household and outdoor products; therefore, human exposure is unavoidable. In this review, we summarized the potential mechanisms of FRs-associated immune and inflammatory signaling and their possible contribution to the development and progression of NAFLD, with an emphasis on FRs-mediated interferon signaling. Knowledge gaps are identified, and emerging pharmacotherapeutic molecules targeting the immune and inflammatory signaling for NAFLD are also discussed.

## 1. Introduction

**Figure 1:**
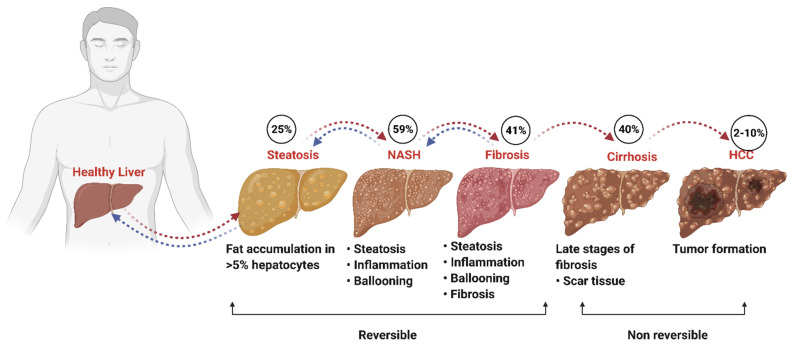
Progression of NAFLD. Simple steatosis is the initial phase of NAFLD characterized by excessive accumulation of fat in the hepatocyte. With time, steatosis progresses to a more inflammatory state called NASH in approximately 59% of patients. In addition, 41% of patients can develop more severe conditions such as fibrosis and cirrhosis (40%), leading to hepatocellular carcinoma in 2–10% [1]. Steatosis, NASH, and fibrosis are reversible with timely and appropriate interventions, while later stages cannot be reversed. (Created with BioRender.com).

Nonalcoholic fatty liver disease (NAFLD) is a significant cause of concern worldwide; it is estimated that about 25% of the world’s adult population suffers from NAFLD [1,2]. NAFLD is highly prevalent in North America, South America, Asia-Pacific, the Middle East, and European countries [3]. The estimated incidence of NAFLD is more than 64 million individuals in the United States and 52 million in the four main European countries (United Kingdom, Italy, Germany, and France) with significant economic costs [4]. NAFLD is characterized by hepatic steatosis, a state of uncontrolled supraphysiological accumulation of fatty acids or triglycerides in the liver [5]. Triglycerides and cholesterol esters stored in the liver are later packed into lipid droplets localized in the cytoplasm [6,7]. The increased lipids species further promote cellular stress and hepatocellular injury, promoting NALFD development to more severe conditions. NAFLD consists of broad-spectrum pathologies; steatosis usually occurs as the first stage of liver damage. With the advancement of time, steatosis typically progresses to a more severe condition such as nonalcoholic steatohepatitis (NASH), fibrosis, cirrhosis, and eventually leads to hepatocellular carcinoma (HCC) [8]. (Figure 1).

Clinically steatosis is distinguished by the intrahepatic accumulation of fat in more than 5% of hepatocytes, whereas NASH is a stage within the spectrum of NAFLD and is distinguished by the presence of steatosis, hepatocellular injury, hepatocyte ballooning, and inflammation with or without fibrosis [9]. The clinical diagnoses of these conditions are difficult because of the lack of diagnostic tools. Current diagnosis mostly relies on liver biopsy, along with certain serum biomarkers such as alanine aminotransferase (ALT) and aspartate aminotransferase (AST), as well as liver imaging [10,11]. Several factors are responsible for the pathogenesis and progression of NAFLD, such as diet, obesity, gut microbiota, genetic predisposition, epigenetic factors, oxidative stress, metabolic factors, and hormonal dysregulation, resulting in altered lipid homeostasis, which leads to lipid accumulation and inflammation [12,13].

Increasing evidence suggests a plausible role of environmental risk factors, including exposure to endocrine and/or metabolism-disrupting chemicals that can alter the function of endocrine signaling in the liver, which is a central organ for controlling lipid homeostasis [14,15]. Moreover, environmental contaminants can influence immune responses by impairing the immune system’s components and, ultimately, disease susceptibility [16]. It has been well recognized that occupational and environmental chemical exposures are associated with the development and progression of NAFLD [17]; therefore, some researchers also propose to use the term TAFLD to describe the spectrum of toxicant-associated fatty liver diseases that may include steatosis, steatohepatitis, cirrhosis, and liver cancer [18]. However, TAFLD was initially used to describe the extent of pathological liver damage similar to alcoholic liver disease and NAFLD following xenobiotic exposure (e.g., prescription drugs) [19], whereas toxicant-associated steatohepatitis (TASH) is the more severe form of fatty liver disease and may arise from exposures to industrial chemicals, such as vinyl chloride, even in the absence of other risk factors for fatty liver such as alcohol consumption, obesity, etc. [20]. However, we are using the well-accepted term NAFLD for further discussion in this paper.

Flame retardants (FRs) are anthropogenic chemicals or mixtures of chemicals widely used in commercial and consumer products such as building materials, automobiles, plastics, textiles, furniture, mobile phones, and toys since the 1960s to reduce the flammability and risk of fire [21,22,23]. The production and use of FRs are steadily increasing globally; in 2008, global production and use were 1.95 million tons, which increased to 2.62 million tons in 2014 [24]. The global market of FRs had a value of USD 6.29 billion in 2015, and it is projected to be USD 11.96 billion by 2025 [25]. The production of organophosphorus flame retardants (OPFRs), which are used as a replacement of largely restricted brominated FRs, has drastically increased over the last few years [26,27].

Biomonitoring studies have demonstrated the presence of FRs in indoor and outdoor environments, including air, house dust, drinking water, sediment, biota [28,29,30], and even in composite food samples [31]. Moreover, FRs and their metabolites are consistently detected in human tissues, urine samples, hair, nails, placenta, breast milk, body fluids, serum, and blood samples indicating exposure to the human population [32,33,34,35,36,37,38]. With the high usage and ubiquitous presence of FRs in various household and outdoor products, human exposure to FRs are unavoidable. Higher exposure to FRs among children, compared to adults, has been reported in a biomonitoring study [39]. The routes of exposure of FRs to humans depend upon the chemical and predominantly include inhalational, ingestion, and dermal routes [40,41,42].

Some occupations, e.g., electronic waste (e-waste), recycling, air transportation, and manufacturing facilities, have been shown to expose workers to high concentrations of FRs [43]. Recently, it has been shown that occupational exposure to OPFRs, polybrominated diphenyl ethers (PBDEs), and novel brominated flame retardants occur in a variety of professions and activities such as chemical manufacturing, carpet installation, foam manufacturing, electronic scrap, rigid board installation, gymnastics, nail salons, roofing, and polyurethane foam application [44]. FRs are a growing concern to human health and the environment and have been identified as high priority chemicals, e.g., in the pan-European initiative Human Biomonitoring for Europe (HBM4EU) [45].

Several health hazards associated with chronic exposure to legacy PBDEs and other FRs used as substitutes have been reported, including developmental, neurotoxicity, reproductive disorders, carcinogenicity, and endocrine and metabolic disruption [28,46,47,48,49,50,51,52]. The in vitro and in vivo endocrine disruption effects of several FRs have been well documented, and studies indicate that FRs could modulate several nuclear receptors (NRs) [53,54,55]. It has been known that the modulation of endocrine signaling alters developmental, reproductive, neurological, and immune functions [56]. Effects of endocrine-disrupting chemicals can occur at very low levels, and early exposures during the development period have been linked with an increased incidence of metabolic-related adverse effects such as obesity, diabetes, and NAFLD [57]. Increasing evidence also indicates an association between exposure to FRs and metabolic syndrome in few epidemiological studies [58,59,60,61]. Therefore, it is important to evaluate FRs-associated risk of metabolic effects such as NAFLD development and progression, especially in occupationally exposed populations and individuals with other risk factors such as obesity and type 2 diabetes (T2D). This review aims to describe possible biological mechanisms altered by FRs in the context of NAFLD, particularly focusing on the role of IFN and inflammatory signals in metabolic disruptions. An outlook on the emerging potential therapies targeting immune and inflammatory signaling for NALFD is also discussed.

## 2. Pathobiology of NAFLD: Role of IFN and Inflammatory Signaling

NAFLD is a complex metabolic disease modulated by numerous factors and manifested with several metabolic alterations [62]. Various biochemical processes portray the pathophysiology of NAFLD, and multiple mechanisms have been proposed. The “two-hit hypothesis” pathogenesis of NAFLD starts with the first hit, i.e., the accumulation of excessive triglycerides in the liver leading to steatosis, and the second hit includes several factors such as oxidative stress that initiate the progression of steatosis to a more severe condition such as NASH [63,64]. In contrast, the “multiple-hit hypothesis” considers multiple parallel events acting synergistically, such as lipotoxicity, adipose tissue inflammation, and gastrointestinal events, including effects on gut microbiota in the development and progression of NAFLD [65,66]. In general, lipid accumulates in the liver as a result of increased de novo fatty acid synthesis, decreased fatty acid oxidation, increased fatty acid influx from peripheral organs to the liver, and decreased fatty acid efflux from the liver [67,68]. It has been suggested that the hepatic accumulation of triglycerides in the liver might be protective toward progressive liver damage [69,70]. However, it is worth noting that NAFLD is a complex disease, and there are lines of evidence that inflammation may precede steatosis and contribute to the advancement of the disease or may even further lead to lipid accumulation in hepatocytes (steatosis) [65]. Moreover, recent findings suggest the involvement of pro-inflammatory cytokines and innate immunity signaling in the pathophysiology of NAFLD, regulating all features of NAFLD progression, including disbalance in lipid homeostasis, metabolic dysregulation, inflammation, and fibrosis [71,72,73,74,75].

Interferons (IFNs) are a type of cytokines that are released/produced as a defense mechanism against viral infections [76] and/or in response to the damage-associated molecular patterns (DAMPs) and pathogen-associated molecular patterns (PAMPs). DAMPs refer to a set of intracellular signaling molecules that are secreted upon cellular stress, injury, or cell death. DAMPs are detected by pattern recognition receptors (PRRs) such as Toll-like receptors (TLRs), NOD-like receptors (NLRs), receptor for advanced glycation end products (RAGE), and c-GAS (a cytosolic PRRs), which activate downstream signaling pathways to induce production of cytokines that drives the progression of NAFLD [77,78,79]. IFNs are classified based on their receptor and downstream signaling cascades into three main classes: type I (IFN-α/β), type II (IFN-γ), and type III (IFN-λs). IFN-α and IFN-β bind to a specific heterodimeric membrane receptor on the cell surface called the IFN receptor (IFNAR), composed of two IFNα and IFNβ receptor subunits (IFNAR1 and IFNAR2). The binding of IFN-α/β to their receptor initiates the downstream signaling and recruitment of receptor-activated protein kinases such as Janus kinase 1 (JAK1) and tyrosine kinase 2 (TYK2), which phosphorylate and activate the transcription factors signal transducer and activator of transcription (STAT1) and STAT2. The phosphorylated STATs recruit IFN regulatory factor 9 (IRF9), which together (STAT1–STAT2–IRF9) forms a complex called IFN-stimulated gene factor 3 (ISGF3). The ISGF3 translocates to the nucleus and binds with IFN-stimulated response elements (ISREs) to initiate transcription of several IFN-related signature genes [80,81]. The dimeric IFN-γ receptor consists of the interferon-gamma receptor 1 (IFNGR1) and IFNGR2 and activates JAK1 and JAK2, which exclusively activate STAT1 [82]. IFN-λ binds to a heterodimeric receptor comprised of two different chains—IFN-λR1 and IL-10R2—leading to the activation of JAK1 and TYK2, which then phosphorylate and activate STAT1 and STAT2. The phosphorylated STATs with IRF9 (ISGF3) enter the nucleus and drive the transcription of IFN-stimulated genes (ISGs). Both type I and type III IFNs activate ISGF3 and therefore induce similar transcriptional responses [83].

The potential role of IFNs in NAFLD has been demonstrated by an increased expression of stimulator of IFN genes (STING) in high-fat diet (HFD)-induced NAFLD mouse model and free fatty acid-induced NAFLD in the human fetal hepatocytes cell culture, whereas knocking down either STING or IRF3 reduced the lipid accumulation, hepatic inflammation, and apoptosis in liver of mice [84]. A higher level of STING was found in liver tissues from NAFLD patients, in mice model of NAFLD, STING-induced macrophage-mediated liver inflammation and fibrosis, whereas STING deficiency attenuated steatosis, fibrosis, inflammation, and insulin resistance in the liver of mice [85]. Activation of STING was associated with an aggravated expression of several inflammatory cytokines, including IL-18, IL-6, IL-1β, TNFα, and C-X-C motif chemokine ligand 10 (CXCL-10) in HFD-induced NAFLD mice, whereas repressing STING signaling attenuated lipid accumulation and liver inflammation [86]. STING is an endoplasmic reticulum-bound protein that induces expression of type I IFNs (IFN-α and IFN-β) [87,88], IRF3, and NFκB through the TBK1 pathway [89]. IRF3 is a transcriptional regulator of inflammation and inflammatory response and has been linked with insulin resistance, as shown in murine adipocytes [90]. Translocation of IRF3 into the nucleus further induces the transcription of type I IFN on binding to ISREs [91].

Considering the expression and involvement of the STING pathway in clinical and experimental models of NAFLD, which led to the induction of IFNs or IRFs, it is hypothesized that IFNs might be a key player in the pathogenesis and progression of NAFLD. More recently, Ghazarian et al. also demonstrated the potential role of IFNs in NAFLD by the finding that IFNαR1−/− mice lacking IFNAR1 were protected from HFD-induced hepatic steatosis and insulin resistance, whereas wild-type mice developed severe NAFLD phenotypes [92]. Moreover, a choline-deficient diet-induced NAFLD mice model showed increased STAT1 and IFN-regulated genes compared to controls, further suggesting a possible role of IFN signaling in NAFLD [93]. It has been observed that the hepatocyte-specific deletion of IFNαR1 worsened steatosis and inflammation but not insulin resistance in mice fed with a choline-deficient diet or HFD. Adipocyte-specific deletion of IFNαR1 worsened metabolic dysregulation and increased insulin resistance but not steatosis. IFNαR1 deletion in myeloid or intestinal epithelial cells was not susceptible to metabolic dysregulation or liver damage [94]. These observations further suggest a diverse tissues specific role of IFN signaling in metabolic dysregulation, which warrants further investigation. Moreover, IRF7, the principal regulator of IFN, increased triglyceride, cholesterol, free fatty acid, and induced lipid accumulation in HFD-fed mice, while knockdown of IRF7 ameliorated diet-induced hepatic steatosis and improved glucose and lipid homeostasis [95].

The downstream mechanisms by which IFN signaling modulates hepatic steatosis or NAFLD progression are still uncertain. However, IFN mediated cytokine production, modulation of receptor responses, transcriptional regulation of IFN target genes, or lipid/glucose metabolism-related genes, inducing lipogenesis and lipolysis, can be involved. Experimental studies demonstrated that IFN-γ-induced lipogenesis in renal mesangial cells by enhancing the expression of high mobility group box 1 (HMGB1), which further upregulated the expression of sterol regulatory element-binding protein 1c (SREBP-1c) and fatty acid synthase via JAK2/STAT1-mediated pathway in mouse [96]. IFN-α stimulated hepatic fatty acid synthesis, increased activity of enzyme acetyl-CoA carboxylase (ACC), which plays an essential role in the fatty acid synthesis, and showed synergistic activity when administered in combination with other cytokines such as TNF or IL-1 in mice [97]. A recent study also indicated the involvement of IFNα in obese-related NAFLD patients, as evidenced by increased IFN-α serum levels that were associated with intramuscular fat in obese patients with NAFLD [98]. Another study also found stimulation of hepatic lipid and cholesterol synthesis after administering various cytokines, including IFNs to mice [99]. These data suggest an important role of cytokines and IFNs in regulating lipid metabolism and inducing metabolic disturbances.

Both type I and type II IFN (IFN-β and IFN-γ) have been reported to induce insulin resistance in vitro in mouse adipocytes by inducing different isoforms of suppressor of cytokine signaling (SOCS) [100]. SOCS plays a significant role in metabolic disease by modulating insulin and pro-inflammatory cytokine signaling. SOCS-1 and SOCS-3 induced insulin resistance by inhibiting phosphorylation of insulin receptors IRS-1 and IRS-2 and downstream signaling in mice liver [101]. Inhibition of SOCS-1 and SOCS-3 in obese diabetic mice improved insulin sensitivity, regulated the expression of SREBP-1c, and ameliorated hepatic steatosis and hypertriglyceridemia [102]. In cultured mouse adipocytes, IFN-α and IFN-γ induced lipolysis and impaired lipoprotein lipase activity [103,104]. IFN-γ increased triacylglycerol and lipid droplets levels in pancreatic β-cells, increased de novo lipogenesis, impaired mitochondrial fatty acid oxidation, and increased expression of lipid metabolism genes via JAK-dependent signaling [105]. Moreover, IFN-γ deficiency attenuated steatohepatitis and fibrosis in MCDHF diet-induced NASH in IFN-γ deficient mice by inhibiting macrophage or Kupffer cell infiltration, inflammatory responses, and hepatic stellate cell activation [106].

However, hepatocyte-specific deletion of JAK2 in mice developed steatosis, whereas the same mice were completely protected against the development of diet-induced steatohepatitis and glucose intolerance [107]. Sos et al. also demonstrated spontaneous steatosis development in hepatocyte-specific JAK2 deleted mice [108]; however, JAK2 hepatocyte-specific deletion protected against ROS-induced oxidative damage [109]. The above studies strengthen the involvement of IFN or IFN regulatory pathways in NAFLD development; however, further studies are needed to explore the potential role of IFNs in the development and progression of NAFLD.

Inflammatory cytokines such as IL-1α, IL-1β, and TNFα play an essential role in lipid metabolism and metabolic diseases [110,111,112]. IL-1 signaling upregulated fatty acid synthase and lipogenic gene expression in obese mice and induced de novo fatty acid synthesis and hepatic inflammation [113]. Kupffer cell-derived inflammatory markers act as major modulators of peroxisome proliferator-activated receptor (PPAR) expression and activity in mice and human primary hepatocytes culture. Kupffer cell-secreted IL-1β suppressed PPARα activity and thereby inhibited fatty acid oxidation, resulting in hepatic lipid accumulation in obese mice [114]. Depletion of Kupffer cells improved hepatic steatosis, suggesting a critical role of Kupffer cells and derived cytokines such as IL-1 in regulating lipid metabolism. IL-1α and IL-1β have been reported to impair insulin signaling by altering tyrosine phosphorylation of insulin receptor substrate (IRS-1 and IRS-2), which leads to insulin resistance [115]. Insulin resistance is one of the prominent and pivotal factors responsible for the development of NAFLD. Insulin resistance increases de novo lipogenesis by increasing SREBP-1c, a transcription factor that activates fatty acid synthesis and lipolysis in the peripheral tissue, ultimately increasing the accumulation of triglyceride in the liver [116,117,118,119,120]. Moreover, insulin resistance leads to inadequate suppression of gluconeogenesis and contributes to hyperglycemia and hepatic steatosis. Under the influence of hyperglycemic condition, the liver transcription factor carbohydrate responsive element-binding protein (ChREBP), which regulate various lipogenic gene expression stimulates glycolysis and lipogenesis in the liver [121]. Insulin resistance also promotes peripheral lipolysis in adipose, subsequently increasing lipid delivery and accumulation in the liver, thereby increasing steatosis [122]. TNFα also contributes to insulin resistance by inhibiting insulin receptor signaling [123,124]. Nevertheless, IL-17A has also been associated with hepatic steatosis and pro-inflammatory response in NAFLD, which facilitated steatosis progression to steatohepatitis with increased inflammation [125]. Together, IFN and inflammatory signaling play a critical role in the pathogenesis and progression of NAFLD. However, further studies are warranted to understand the molecular mechanisms and clarify the role of JAK–STAT and tissue-specific role of IFN signaling pathway in the pathophysiology of NAFLD.

## 3. Biological Actions of FRs: Role in Inflammatory and Cytokine Signaling

**Figure 2:**
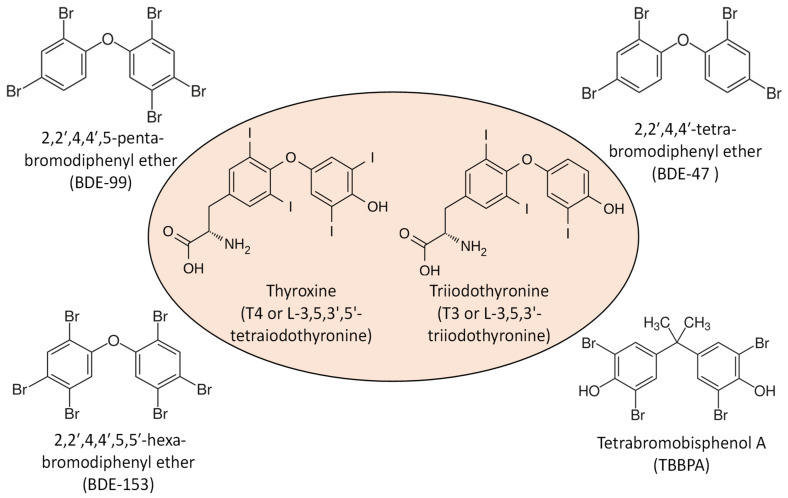
Structure similarity between thyroid hormone [thyroxine (T4) and triiodothyronine (T3)] and flame retardants (PBDE, e.g., 2,2′,4,4′,5-Pentabromodiphenyl ether (BDE-99), 2,2′,4,4′-Tetrabromodiphenyl ether (BDE-47), 2,2′,4,4′,5,5′-Hexabromodiphenyl ether (BDE-153), and novel brominated flame retardant, e.g., Tetrabromobisphenol A).

FRs are associated with various pathological alterations, as documented in Table 1. Many FRs, such as PBDEs, are structurally close analogs of thyroid hormones (THs), e.g., thyroxine (T4) and triiodothyronine (T3) (Figure 2); therefore, considerable evidence suggests a role of PBDEs-mediated thyroid-associated endocrine disruption in animals and humans [126,127,128,129,130,131,132]. THs are crucial for development, growth, and metabolic activity, and their importance in hepatic fatty acid and cholesterol synthesis, including metabolism, has been well documented [133]. Growing evidence also points toward the induction of reactive oxygen species (ROS) or oxidative stress biomarkers by FRs, which may contribute to their toxicity, endocrine disruption effects [134,135,136], and metabolic dysregulation [137,138]. For example, short-term exposure to environmentally relevant doses of dechlorane plus showed endocrine disruption effects in zebrafish and increased hepatic catalase activity, a marker of oxidative stress [139]. PentaBDE exposure to rats disturbed redox homeostasis, intensified lipid peroxidation, and induced symptoms of fatty liver disease [140]. Exposure to penta-BDE mixture of BDE-99 and BDE-100 caused histopathological changes and hepatocellular injury in rat liver tissue, increased liver mass, fatty degeneration, hepatocytes hypertrophy, and vacuolization [141,142].

DBDPE affected liver function parameters, such as ALT and AST levels, and caused hepatocyte hypertrophy and cytoplasmic vacuolization in mice liver and induced hepatic cytochrome P450 (CYP450) enzymes such as CYP1A, CYP2B, and uridine diphosphate-glucuronosyltransferase [143]. These enzymes are downstream activators of nuclear xenobiotic receptors (NXR), such as aryl hydrocarbon receptor (AhR) and chimeric antigen receptor (CAR). Perinatal exposure of human-relevant doses of 2,2′,4,4′-tetrabromodiphenyl ether (BDE-47) to rat showed differential expression of genes encoding for various biological process and pathways, including activation of the CYP450 in the pathways of PXR/RXR, and CAR and metabolic pathways of lipid, carbohydrate and amino acid, cofactor, and vitamin metabolism in the liver tissues of rat offspring [144].

DE-71, a mixture of PBDE-induced liver histopathological alterations such as hepatocellular hypertrophy, vacuolization, and necrosis in rats, CYP1A and CYP2B induction indicated NR activation, i.e., AhR and CAR [145]. In human liver cell culture, several OPFRs caused intracellular lipid accumulation through increased fatty acid biosynthesis, inhibition of β-oxidation, increased de novo fatty acid, triglyceride, and cholesterol synthesis, as well as mitochondrial dysfunction [146]. Maternal exposure to OPFR mixture including tris(1,3-dichloro-2-propyl) phosphate, tricresyl phosphate, and triphenyl phosphate (TPhP) to mice altered the expression of various genes related to fatty acid synthesis, glucose, and triglycerides metabolism in the liver of mice offspring. It was suggested that the effects were possibly mediated by modulation of NRs, including estrogen receptor (ERα), PPARγ, insulin receptor, ghrelin receptor, and leptin receptor [147].

Exposure of human liver cell culture to 9,10-dihydro-9-oxa-10-phosphaphenanthrene 10-oxide (DOPO) affected various genes and pathways representing several biological processes, including fatty acid metabolism and glucose transport, along with cellular stress response pathways [148]. Furthermore, neonatal exposure to TPhP and its metabolite diphenyl phosphate (DPHP) induced sex- and dose-dependent metabolic disruptions in adult mice. Low dose exposure to TPhP upregulated lipid-related metabolites in serum of male mice while exerted no significant effects on female mice. High doses downregulated the pyruvate metabolism and citric acid cycle pathway, reflecting abnormal lipid metabolism after TPhP exposure in male mice [149].

Moreover, in utero, exposures of rat dams to DE-71 and BDE-47 decreased T4 level and upregulated hepatic transcripts of CYPs and conjugation enzymes, Nrf2, and ATP-binding cassette (ABC) transporters in postnatal day four (PND 4) rat pups, indicating metabolic alterations and increased oxidative stress. Cytoplasmic vacuolization in the liver was also observed in DE-71 exposure groups [150]. Exposure to a human-relevant dose from the dust of a commercial mixture of FR Firemaster^®^ 550 has been reported to be a potential obesogen, endocrine disruptor, and contributed to metabolic syndrome and insulin resistance in rats [151].

A direct effect of FRs on cytokine production was reported in numerous studies. DE-71 enhanced pro-inflammatory response and increased secretion of inflammatory cytokines such as interleukin (IL)-1β, IL-2, IL-4, IL-6, IL-8, IL-10, IL-17A, IL-17, TNF-α, and IFN-γ in vitro in human peripheral blood mononuclear cells (PBMCs) [178]. BDE-47 induced ROS and stimulated pro-inflammatory cytokine release in first-trimester human extravillous trophoblast cell culture [179]. Hexabromocyclododecane (HBCD) and tetrabromobisphenol A (TBBPA) have been reported to alter the secretion of inflammatory cytokines such as TNFα, IL-1β, and IFN-γ through mitogen-activated protein kinase (MAPK)-associated extracellular-signal-regulated kinase (ERK1/2) pathways in human PBMCs [180,181]. HBCD significantly increased intercellular adhesion molecule (ICAM-1), IL-6, and IL-8, whereas TBBPA significantly increased the expression of ICAM-1 and IL-6 in human bronchial epithelial cell culture [182]. Involvement of NR signaling in TBBPA-mediated inflammatory response was reported, which might have stimulated the JAK–STAT signaling in bronchial epithelial cells.

TBBPA is a halogenated analog of bisphenol A (BPA). BPA is a widely used chemical in several products and has been known to show numerous adverse effects besides well-known xenoestrogen effects. BPA exposure has been identified as a risk factor for metabolic disease, including NAFLD, and epidemiological studies find an association between BPA exposure or urinary BPA levels with the NAFLD [183,184]. However, the analogs of BPA- TBBPA and Tetrachlorobisphenol A (TCBPA) are found to be more cytotoxic to the rat hepatocytes than BPA itself and induced oxidative stress and mitochondrial dysfunction and decreased ATP production [185]. TCBPA treatment of mice significantly induced secretion of various pro-inflammatory cytokines (IL-2, IL-12, TNF-α, and IFN-γ) and immunosuppressive cytokines (IL-4, IL-5, IL-10, GM-CSF) in serum, suggesting immunosuppressive property of TCBPA [186]. Moreover, the transcriptomic analysis revealed that TBBPA induced changes in expression levels of transcripts associated with hepatic IFN pathway regulation and genes regulating fatty acid metabolism in rats [187]. TBBPA has also been shown to induce adipogenesis (lipid accumulation) in mouse-derived preadipocytes cell culture [188].

Recently, tetrabromo-ethyl cyclohexane (TBECH) has also been reported to exhibit immunotoxic property, enhanced lactate dehydrogenase, and upregulated expression of IL-1β, IL-6, and TNF-α, proapoptotic genes, antigen presenting-related genes as well as induced oxidative stress in vitro in murine macrophages [189]. Similarly, decabrominated diphenyl ethers (BDE-209) and DBDPE-induced inflammation and upregulated various inflammatory mediators IL-1β, IL-6, IL-10, and TNFα in serum samples of male rats [190]. Moreover, perinatal exposure to low doses of BDE209 increased the pro-inflammatory cytokines IL-4, IL-6, IL-10, TNFα, IFN-γ, and IL-17 in the serum of male offspring [191]. BDE209 also promoted TLR4-dependent lipid uptake and enhanced lipid accumulation in vitro in human macrophages [192].

It has recently been shown that the six common OPFRs (TPHP, TDCPP, TNBP, TOCP, TCEP, and TBOEP) affected inflammation-related pathways, including JAK–STAT, TNF signaling, and PI3K–Akt pathways in varying degrees in vitro in human macrophages [193]. The presented data suggest that numerous FRs are capable of inducing cytokines and inflammatory mediators, which might interplay with other signaling pathways.

Elevated levels of cytokines, including TNF-α, IL-lβ, IL-6, and chemokine monocyte chemoattractant protein-1 (MCP-1) after BDE-47 exposure, induced liver inflammation in mice [194], aggravated hepatic steatosis and fibrosis in the mouse by oxidative stress and increased pro-inflammatory cytokines [152]. BDE-209 and DBDPE induced liver morphological changes by oxidative stress and inflammation by increasing levels of TNFα and IL-6 [153]. BDE-209 has been reported to induce mitochondrial dysfunction in isolated rat liver and increased ROS accumulation in human liver, induced cytochrome c release, and apoptotic cell death [195]. Acute exposure to tris (1,3-dichloroisopropyl) phosphate (TDCIPP) significantly upregulated the expression of inflammatory genes IL-1β, IL-6, IL-10, IL-12a, IL-13, IL-15 IL-26, including TLR signaling pathways such as TLR8a, TLR8b, TLR9, AP1, STAT1b, and IRF7, leading to inflammation and hepatotoxicity in zebrafish [196]. Therefore, the collected evidence suggests a plausible role of FRs and their metabolites in hepatotoxicity or steatosis through inflammatory cytokines or IFN-dependent pathways. Together, these findings support the FRs-mediated interplay between cytokines and oxidative stress in the etiology of NAFLD. Table 2 summarizes the literature evidence of FR-mediated induction of IFN or IFN components that are associated with the IFNs role in NAFLD.

## 4. Modulation of IFN Signaling by FRs and Role in NAFLD

A growing body of evidence suggested that FRs and/or their metabolites modulated the inflammatory and cytokines signaling, including the immunological pathways in several in vitro and in vivo studies. However, direct studies reporting FRs mediated IFN modulation leading to NAFLD is scarce, although ample evidence exists that strengthens FRs’ role in cytokine modulation, as presented in Table 2. Among the critical regulators of immune responses and inflammation, including IFN signaling, are the mitochondria, which contain approximately 1000 distinct proteins [208,209]. Mitochondria are involved in cellular respiration and ATP synthesis; however, apart from the canonical actions, a vast range of biological processes including energy homeostasis, metabolism, signaling, inflammation, and immune functions are also regulated by mitochondria [210,211,212]. Lipophilic characteristics of mitochondrial membranes facilitate the accumulation of lipophilic compounds, and mitochondrial DNA (mtDNA) is potentially more prone to be affected by the lipophilic environmental toxicants [213,214]. Moreover, mounting evidence also reports FRs-induced mitochondrial dysfunction in several in vitro and in vivo studies [195,215,216,217], and recently, biomarkers of mitochondrial dysfunction have been positively correlated with lipid accumulation potential of several OPFRs in mouse hepatocytes culture [218]. Observations in patients and animal models of NAFLD/NASH demonstrated mitochondrial dysfunctions, as often observable characteristics in NAFLD [219,220,221].

**Figure 3:**
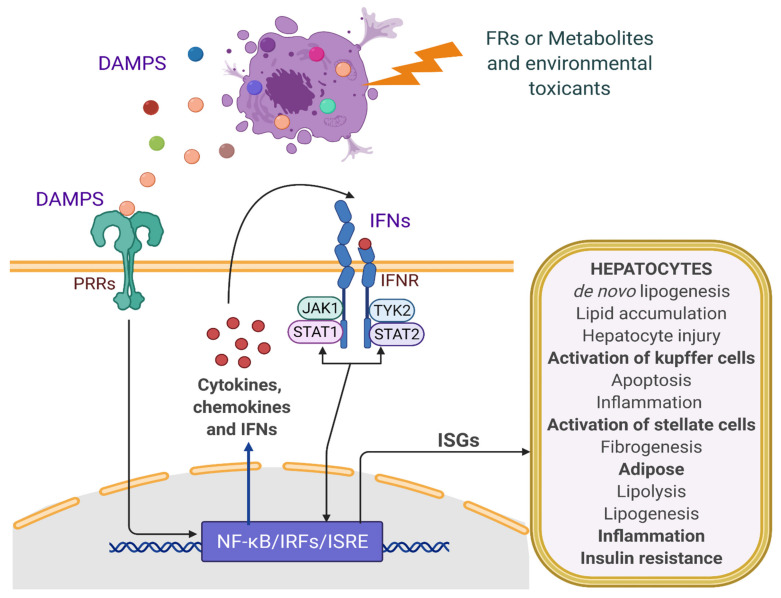
Scheme showing role of FRs-mediated cytokine and IFNs production and potential contribution in the progression of NAFLD. DAMPS are produced by damaged cells and bind to PRRs such as TLRs and NLRs, or cytosolic DNA sensors (cGAS), resulting in downstream signaling leading to activation of inflammatory mediators, cytokines, and IFNs. IFNs function through the respective IFN receptors and downstream JAK–STAT signaling, leading to the expression of interferon stimulatory genes (ISGs), which modulate many biological processes involved in the progression of NAFLD. (Created with BioRender.com).

**Figure 4:**
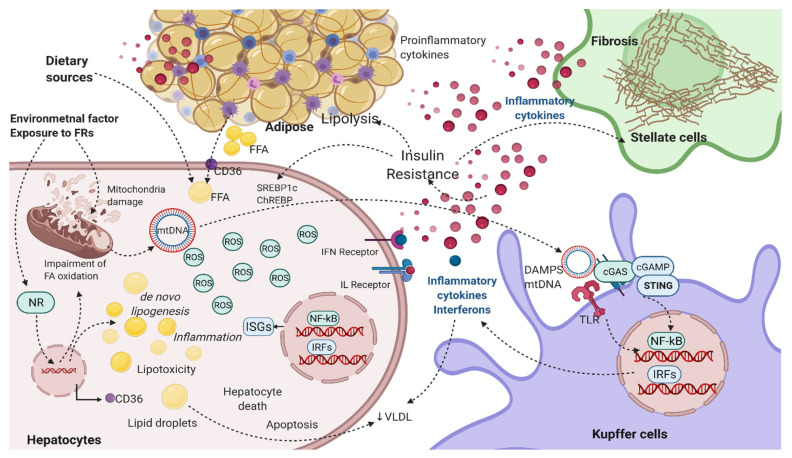
Schematic of FRs-mediated proposed mechanisms and pathways involved in the pathophysiology and progression of NAFLD. FRs, through several distinct mechanisms, could cause biochemical disruptions of many metabolic processes. FRs can induce direct mitochondrial damage or impair the mitochondria function and β-oxidation, thereby inducing ROS and inflammatory signaling. Alternatively, FRs could activate the NXRs. Since NRs are the central regulators of hepatic lipid metabolism, activation of NXR such as PXR could increase lipogenic gene expression, leading to increase de novo lipogenesis, inhibition of fatty acid β-oxidation, and increases in fatty acid import through upregulation of CD36. The impairment of mitochondrial β-oxidation induces the long-chain fatty acids metabolism via peroxisomal β-oxidation and ω-oxidation in the cytochromes. These processes further generate a considerably high amount of ROS, promoting oxidative stress, in turn inducing damage to the mitochondrial membranes, compromising cellular respiration and metabolism, and impairing liver function by cellular damage. Damaged mitochondria release the mtDNA into the cytosol, where it gets recognized as DAMPs by several PRR such as TLR or cGAS. cGAS is an innate immune sensor, which generates a second messenger cGMP and activates STING by translocating it to the perinuclear Golgi complex and serves as a signal for TBK1 and IKK. This promotes the phosphorylation and nuclear translocation of IRF3 and NF-κB inhibitor IκBα, and stimulation of IFN, whereas NF-κB translocation to the nucleus activates pro-inflammatory cytokines. In comparison, TLR activates either MyD88-dependent or TRIF-dependent signaling pathways and induces the expression of various inflammatory cytokines (TNFα, IL-1β, IL-6, IFNs). Cytokines, e.g., IFNs, bind to their respective receptors and initiate the downstream signaling, which phosphorylates and activates the transcription factors and initiates transcription of several IFN-related genes responsible for insulin resistance and activation of inflammatory mediators and de novo lipogenesis. Insulin resistance stimulates hyperinsulinemia, which increases glycolysis and promotes de novo lipogenesis by enhancing ChREBP and SREBP-1c, significantly contributing to lipid accumulation. Insulin resistance increases lipolysis, leading to increased free fatty acids delivery from the peripheral organs into the liver mediated by elevated CD36. Inflammatory mediators, such as IFNs, TNFα, and IL-6, may further decrease VLDL export and facilitate lipid accumulation. The net result is an escalation of hepatic steatosis and inflammatory condition, eventually leading to more severe NAFLD/NASH conditions. (Created using BioRender.Com).

In the case of FRs, the mechanisms associated with the development and progression of NAFLD might be chemical specific; however, exposure to FRs could potentially damage the mitochondria or hamper mitochondrial function. Damage to the mitochondria can release the genomic DNA or mtDNA into the cytoplasm, where it serves as DAMPs and activates the PRR for instance, cGAS–cGAMP–STING pathway, leading to enhanced production of inflammatory cytokine, including IFNs [222,223,224]. Mitochondria damage also generates ROS and pro-inflammatory cytokines, which can further lead to cellular injury. mtDNA could activate other PRRs such as TLR in the Kupffer cells and infiltrating monocytes, thereby increasing the expression of various inflammatory cytokines, including IFNs [225,226]. IFNs can further lead to steatosis and NAFLD progression, possibly by modulating the expression of lipid metabolism-related genes, increasing de novo lipogenesis, increasing lipolysis in the peripheral tissues, insulin resistance, and increasing inflammation and cellular injury, apoptosis, or cell death, as depicted in Figure 3 and Figure 4.

In addition, oxidative stress may contribute to the development of insulin resistance through p38-MAPK- or other stress-activated kinases such as JNK, GSK-3β, and IKKβ-dependent mechanisms [227]. Nevertheless, studies have shown that OPFRs are effectively metabolized or biotransformed in humans [228]. Biotransformation of FRs by CYP450 metabolizing enzymes may produce metabolites with different physicochemical and toxicological properties [229]. Significant associations between PBDE exposure and CYP2E1 mRNA in the placenta and CYP1A1 mRNA in the fetal liver in human subjects were observed [230]. Increased CYP2E1 has been reported to be associated with fatty liver disease. CYP2E1 generates a significant amount of ROS and triggers oxidative damage such as lipid peroxidation and impairment of mitochondrial function and cellular injury through various signaling pathways, including JNK signaling [231]. CYP2E1 by hydroxylation of fatty acid promotes the formation of cytotoxic lipid species that further promote cellular stress and induce hepatocellular injury or cell death [232].

Moreover, FRs such as OPFRs and PBDEs have also been shown to induce CYP450 enzymes and NXR, which may negatively influence redox homeostasis [233,234]. Thus, FRs could directly induce mitochondrial damage by hampering the mitochondrial function, a well-known target of environmental toxicants [235,236,237], or indirectly by CYP450 xenobiotic-metabolizing enzymes that are known to affect redox homeostasis. Exposure to FRs could modulate the NRs, which are reported to directly control the CYP450 responses and transcription of various CYP isoforms [238,239]. Taken together, mitochondrial damage and/or modulation of NRs could be a critical biological factor responsible for FRs-mediated IFN signaling during the development and progression of NAFLD as depicted in Figure 4.

## 5. Role of FRs in Modulating Other Signaling Linked to NAFLD Biology

Hypothyroidism has been closely associated with NAFLD, which is supported by various epidemiological studies. For instance, Guo et al., in a meta-analysis involving 61,548 participants, reported that elevated thyroid-stimulating hormone (TSH) levels were significantly associated with a higher risk of NAFLD [240]. More recently, Tanase et al. summarized the correlation between hypothyroidism and NAFLD, emphasizing the role of the TH-liver axis in lipid and cholesterol metabolism, insulin resistance, oxidative stress, and inflammatory and immune pathogenesis in NAFLD [241]. An increased TSH level after FRs exposure suggested that FRs act as a competitive inhibitor of the thyroid receptor, leading to hypothyroidism [242]. Furthermore, several FRs, including OPFRs and PBDEs, have been reported to exert thyroid receptor β (TRβ) antagonistic activity [243]. Additionally, it has been reported that elevated TSH binds to thyrotropin receptor (TSHR) on hepatocytes and induced hepatic steatosis via the SREBP-1c lipogenic pathway in rodents [244]. TSH induces gluconeogenesis and diminishes hepatic bile acid synthesis, which plays a crucial role in the digestion of dietary fats and regulation of lipid and glucose metabolism, and inflammatory responses [245]. Bile acids are the endogenous ligand of the farnesoid X receptor (FXR) [246]. Bile acid activation of FXR regulates hepatic de novo lipogenesis, gluconeogenesis, glycogenolysis, inflammation and improves insulin sensitivity, as reviewed [247]. Thus, FRs-mediated thyroid deregulation or hypothyroidism could be another connecting link for their involvement in the pathogenesis of NAFLD. TH signals by binding to the liver-specific receptor (THRβ), which plays a central role in the metabolism and utilization of lipids [133]. TR cross talk with metabolic pathways and other transcription factors such as PPARα and activates the expression of genes involved in fatty acid β-oxidation [248,249]. Inhibition of TR by FRs could impair TH signaling and reduce fatty acid utilization resulting in the esterification of fatty acids and triglycerides accumulation in the liver.

Moreover, dysregulations of NRs, including the NR1 subfamily, have been indicated as molecular initiating events (MIEs) in the pathogenesis of NAFLD in the adverse outcome pathways (AOP), leading to hepatic steatosis [250]. The NR subfamily heterodimerizes with retinoid X receptor (RXR) and regulates the transcription of various genes involved in energy homeostasis, lipid, glucose metabolism, and inflammation [251]. These changes can lead to reprogramming the transcription of genes involved in hepatic lipid homeostasis leading to NAFLD [14,251]. Thus, FRs could act by several distinct pathways causing biochemical disruption and leading to NAFLD and progression to NASH and eventually HCC particularly via modulation of NRs, ROS production, lipid peroxidation, cytokine release, insulin resistance, and mitochondrial dysfunction, as outlined in Figure 4.

It is well known that environmental exposure for chemicals or toxicants is lower, compared to occupational/industrial or accidental exposure. Nevertheless, the duration of environmental exposures (especially during vulnerable lifecycle stages) can be much greater, compared to occupational exposures [252]. Human exposure to FRs can range from various developmental stages, and exposures during early life are especially considered of concern. Environmental exposure can cause a wide range of effects depending on the route and dose of exposure and the individual’s susceptibility, e.g., preexisting disease, age, gender, and genotype. Experimental studies may have been conducted with high doses and may represent high exposure conditions, e.g., following accidental or occupational exposures of FRs. However, epidemiological studies suggest that even low doses (environmental exposure) may cause adverse effects (see, e.g., the recent review [52]).

Various epidemiological studies positively correlate the presence of FRs with oxidative stress, thyroid endocrine disruption, and inflammatory biomarkers. Recently urinary OPFRs metabolite namely dibutyl phosphate (DNBP), bis(1-chloro-2-propyl) phosphate (BCPP), bis(2-chloroethyl) phosphate (BCEtP), bis(1,3-dichloro-2-propyl) phosphate (BDCPP), and DPHP were detected in pregnant women and were positively associated with the oxidative DNA damage– 8-hydroxy-2′-deoxyguanosine (8-OHdG) and lipid peroxidation (8-isoprostane) biomarkers in urine [253]. OPFR metabolites such as dibutyl phosphate (DBP) and DPHP were positively associated with oxidative stress biomarkers, malondialdehyde (MDA), oxidative stress DNA damage biomarkers (8-OHdG), and TSH levels in maternal and neonatal human urine samples [254]. This suggests that oxidative stress and possible thyroid disruption due to OPFRs during pregnancy are of concern.

Another study finds a positive association between the presence of FRs, e.g., (TCPP, TCEP, TNBP, and TPHP) and elevated oxidative stress DNA biomarkers (8-OHdG) in urine samples from participants living in an e-waste dismantling area [134]. A significant positive association was also observed between serum levels of PBDE (BDE-99, BDE-100, and BDE-153) and liver biomarker alkaline phosphatase [255]. These data further suggest a potential involvement of environmental exposure to various toxicants, including FRs, and effects such as oxidative stress, endocrine disruption, inflammation, and hepatic imbalance. Due to the lipophilic nature of FRs, particularly brominated FRs, they can easily penetrate the epithelial barriers and accumulate in fats such as adipose tissues for an extended period and could also be responsible for metabolic syndrome and T2D [58]. Neonatal or maternal exposure to FRs could cause epigenetic modulation, which can manifest into metabolic alteration later in life [147,256,257].

## 6. Emerging Pharmacotherapeutics for NAFLD Targeting Immune or Inflammatory Signaling

With no Food and Drug Administration (FDA)-approved drugs for NAFLD–NASH, current first-line treatment includes managing metabolic syndromes such as hyperlipidemia, hyperglycemia, and obesity with lifestyle modifications, including diet and physical exercise. However, several pharmacological therapies aiming to alleviate NAFLD and NASH are currently being examined at various phases of clinical trials. Currently, ongoing pharmacotherapy trials for NAFLD and NASH focus on a multitude of mechanisms and diverse targets, including insulin resistance, de novo lipogenesis, oxidative stress, targeting nuclear receptor, and immune or inflammatory signaling.

It is interesting to note that targeting immune or inflammatory signaling pathways has shown promising results in preclinical studies. A phase II clinical trial is currently ongoing with an antagonist of TLR4 (JKB-122) (National Clinical Trial number- NCT04255069). TLR4 plays a pivotal role in innate immunity, inflammatory response, and NAFLD pathogenesis. Other therapeutic immune or pro-inflammatory targets include JNK Inhibitor- CC-90001, which is also being evaluated in phase II trial in subjects with NASH and stage 3 or stage 4 liver fibrosis (NCT04048876).

In addition, a phase II study with leronlimab (PRO 140)-humanized monoclonal antibody antagonist to C-C chemokine receptor type (CCR5) was started to evaluate the efficacy in NASH patients (NCT04521114). Meriva^®^, a complex of phosphatidylcholine and curcumin with potent anti-inflammatory property is being evaluated in phase II trials in pediatric NAFLD patients of ages between 8 and 17 years old (NCT04109742). Curcumin acts as a regulator of inflammation by downregulating the expression of various inflammatory cytokines, including TNF-α, IL-1, IL-2, IL-6, IL-8, and IL-12, MCP, and mitogen-activated and JAK–STAT signaling [258]. The ongoing trial with a wide spectrum of molecules targeting different pathways holds promise for the potential use of some molecules in clinical practice.

## 7. Conclusions and Future Perspectives

NAFLD is a multifactorial disease and a growing health problem globally. Dietary factors, host genetics, gut microbiota, innate immunity, and inflammatory signaling contribute to the pathophysiology of NAFLD [259]. Other contributing factors for the onset and progression of NAFLD include obesity, diabetes, and exposure to occupational or environmental toxicants [260]. The environmental or occupational exposure to chemicals such as FRs might increase an individual’s susceptibility to NAFLD. Early life exposures (or during development) might alter the genes involved in hepatic lipid homeostasis, which can be associated with metabolic dysfunction later in life [14,261]. Various FRs such as OPFRs are a concern to human and environmental health because of their increasing levels in the environment and exposure to FRs leads to various biological alterations including metabolic disruption. Besides modulation of NRs and endocrine-disrupting potential, FRs are capable of modulating inflammatory and IFN-mediated signaling. The direct interaction of FRs with STING remains largely unknown. However, the putative molecular mechanisms could include DAMPs mediated activation of cGAS–cGAMP–STING or other PRRs mediated-IFN-signaling pathways. Nevertheless, the biological effects of FRs in NAFLD onset and progression need to be studied using human disease-relevant models.

A better understanding of the tissue-specific role of STING or IFN signaling and their cross talk with other pathways may help to understand the potential mechanisms for the development and progression of NAFLD. Moreover, the underlying mechanism of how environmental factors, including external exposome, moderate the immune signaling such as STING and IFNs pathway needs further investigation. IFN signaling appeared to be involved in NAFLD–NASH, as evidenced by various clinical and experimental studies. Therefore, more mechanistic studies are required to understand the precise role of IFN pathways in NAFLD for potential therapeutic utilization. It is recommended to perform these mechanistic studies using clinically relevant disease models.

## Figures and Tables

**Table 1:** In vitro and in vivo experimental evidence highlighting the hepatotoxic potential of FRs and associated biochemical dysregulations.

Flame Retardants	Inference and Summary	Test System	References
BDE-47	Aggravated hepatic lipid accumulation by upregulating fatty acid synthesis and suppressing lipid exportation and β oxidation. Increased inflammation, oxidative stress, and serum transaminase levels.	C57BL/6J mice fed HFD	[152]
BDE-209, DBDPE	Induced liver histological changes and interfered with lipid metabolism through oxidative stress. Increased γ-glutamyl transferase, glucose, total bilirubin, and indirect bilirubin levels in serum.	Sprague Dawley rats	[153]
BDE-47, BDE-32	Increased several pro-inflammatory genes, induced oxidative stress and DNA damage, and altered mitochondrial function.	human hepatocellular carcinoma cells (HepG2 cells)	[154]
TDCPP, TCPP, TCEP	Increased expression of apoptotic protein and lactate dehydrogenase enzyme. Downregulated antioxidants genes such as superoxide dismutase and catalase.	Human hepatocarcinoma cells (SMMC-7721 cells)	[155]
TDCPP	Induced oxidative stress and cell cycle arrest in the liver. Increased caspase-dependent apoptotic pathways in the liver and induced cellular damage.	Adult zebrafish	[156]
TOCP	Increased serum ALT and AST levels, oxidative stress in the liver, and hepatocellular injury. Inhibited the viability of the mouse liver cancer cells.	Mouse liver cancer cells (Hepa 1–6) and mice	[157]
BDE-153	Induced ROS generation, genomic instability, autophagy, apoptotic cell death by mitochondrial dysfunctions.	HepG2 cells	[158]
OctaBDE	Impaired redox homeostasis and induced oxidative stress in the liver.	Wistar rat	[159]
TCPP	Disturbed cell growth/division, energy metabolism, signal transduction, defense, and stress response. Increased ROS with an increased expression of Bcl-2 family encoding genes.	Human fetal liver (L02 cells)	[160]
DE-71	Induced pathological alterations in the liver, including increased liver weight, hepatocytic hypertrophy, vacuolation, and necrosis. Increased CYP1A1, CYP1A2, CYP2B, thyroid lesions, and decreased serum thyroid hormone (T4) levels in rats.	F344/N rats and B6C3F1 mice	[161]
BDE-47	Elicited ROS production, lipid peroxidation and modulated the mitochondrial membrane potential.	Human fetal liver–derived hematopoietic stem cells	[162]
BDE-209	Increased oxidative stress, serum glucose, insulin, and triglyceride, and induced structural changes in liver and adipose tissue.	ICR mice	[163]
BDE-47	Exposure to environmentally relevant concentrations during development increased lipid uptake and accumulation by upregulating CD36 and altered expression of metabolic genes, possibly by the mTORC1 signaling pathway.	Pregnant CD-1 mice	[164]
HBCD	Altered transcriptomic profiles of xenobiotics metabolism, oxidative stress, immune response, lipid, glucose metabolism, circadian regulation, cell cycle, fibrotic activity, and hormonal regulation in both males and female rats.	Fischer rats	[165]
TDCIPP	Increased pro-inflammatory cytokine and plasma bile acid levels and disrupted lipid homeostasis.	Chicken embryos	[166]
TPhP	Disrupted hepatic carbohydrate, lipid, fatty acid, amino acid metabolism pathway and DNA damage repair system. Induced histopathological damage in the liver.	Adult zebrafish	[167]
HBCD	Enhanced hyperglycemia, hyperinsulinemia, insulin resistance, and hepatic steatosis. Increased adipose tissue inflammation.	Male C57BL/6JJcl mice fed HFD	[168]
Penta & Deca BDPE	Induced liver microsomal enzymes and impaired redox homeostasis. Increased fatty degeneration and microvascular steatosis in the liver.	Female Wistar rats	[140,169]
BDE-47	In utero exposure induced obesity, hepatic steatosis, glucose intolerance by altering lipid metabolism-related genes, and gut microbiome dysregulation. Promoted inflammation, fatty acid uptake, and inhibited fatty acid catabolism.	Pregnant ICR mice	[170]
AMEP, ADEP	Induced mild hepatotoxicity, fatty degeneration, and necrosis of the hepatocytes.	BALB/c mice	[171]
Dechlorane Plus	Induced oxidative stress and DNA damage in the liver. Altered hepatic carbohydrate, lipid, nucleotide, and energy metabolism via MAPK and JAK–STAT signaling.	Mice	[172]
TCEP	Induced hepatotoxicity by oxidative stress, mitochondrial impairment, DNA damage, and affected cellular senescence.	HepG2 cells	[173]
THP	Induced endoplasmic reticulum stress-mediated apoptosis and cell cycle arrest. Induced hepatocyte ballooning, degeneration, and acute liver injury in mice.	L02 cells, mouse hepatocyte (AML12), and C57BL/6 mice	[174]
BDE-99 perinatal exposure	Increased ROS production, induced thyroid hormone disruption and increased body weight of rat pup. Decreased levels of the cell survival PIP3K/Akt pathway and cyclin D1 in rat pup livers.	Sprague Dawley rats	[175]
EHDPP	Affected energy homeostasis, endoplasmic reticulum stress, apoptosis, cell cycle, and inflammation response pathways in cells.	L02 cells	[176]
TBBPA	Induced oxidative stress, mitochondria damage, and apoptosis in the hepatocytes by the Nrf2 pathway.	L02cells	[177]

TCPP, tris (2-chloropropyl) phosphate; EHDPP, 2-ethylhexyl diphenyl phosphate; TCEP, tris (2-chloroethyl) phosphate; TOCP, Tri-ortho–cresyl phosphate; DBDPE, Decabromodiphenyl ether; OctaBDE, Octabromodiphenyl ether; HBCD, hexabromocyclododecane; BDE-47, 2,2′,4,4′-tetrabromodiphenyl ether; BDE-153, 2,2′,4,4′,5,5′-hexabromodiphenyl ether; TBECH, 1,2-dibromo-4-(1,2-dibromoethyl)-cyclohexane; BDE-32, 2,4′,6-tribromodiphenyl ether; BDE-99, 2,2′,4,4′,5-Pentabromodiphenyl ether, ADEP, aluminium diethylphosphinate; AMEP, aluminium methylethylphosphinate; FABP4, fatty acid-binding protein; HFD, high-fat diet; TBBPA, Tetrabromobisphenol A.

**Table 2:** A summary of the role of IFN signaling in the development and progression of NAFLD and FRs-mediated IFN signaling, which can potentially impact NFALD biology.

Contribution of the IFNs Signaling in NAFLD	FRs-Mediated IFN Signaling
Higher frequencies of IFN-γ+ and/or IL-4+ cells were detected among CD4+ T cells in peripheral blood of NASH patients [197]. Increased IFN-γ in the liver of pediatric (<15 years) NASH patients was observed [198]. IFN-γ induced liver inflammation, hepatocyte injury in the progression of NASH in mice [106].	DE-71 enhanced IFN-γ in vitro in PBMCs [178]. Prenatal exposure to decabrominated diphenyl ether (DBDE) increased IFN-γ in the bronchoalveolar lavage fluids in offspring mice [199].
IFN-γ contributed to hepatic inflammation in diet-induced NASH in rats, rat macrophage, and hepatocellular carcinoma cell lines [200].	TBBPA increased IFN-γ in vitro in human PBMCs [180,181].
IFN-γ-treatment activated hepatic stellate cells and increased hepatocyte apoptosis, hepatic inflammation, serum AST and fibrosis in mouse liver [201].	TCBPA increased secretion of IFN-γ in the serum of mice [186].
STING-IRF3 activation-induced inflammation, hepatocyte injury and apoptosis, and disturbed glucose and lipid metabolism in mice and in LO2 cells [84].	BDE209 increased IFN-γ in the serum of male offspring [191].
Type I and/or type II IFN signaling was associated with oxidative damage in mouse hepatocytes [202] as well as insulin resistance in mouse adipocytes culture [100].	TPHP, TDCPP, TNBP, TOCP, TCEP, and TBOEP modulated JAK–STAT signaling in human leukemia monocytic culture [193].
TNF-α and type I IFN production in Kupffer cells and dendritic cells induced hepatic cell death leading to NASH in mice and murine normal hepatocyte cell culture by TLR7-mediated signaling [203].	TDCIPP upregulated TLR signaling, STAT1, IRF7, and induced inflammation and hepatotoxicity in zebrafish [196].
Upregulation of IL-1β, TNF-α, and IFN-γ in the liver of mice in NASH [204,205].	TBBPA upregulated hepatic IFN signaling and genes regulating fatty acid metabolism in rats [187].
Increased TLR4 and IRF3 gene expression were observed in patients with NASH and hepatocytes exposed to palmitate and lipopolysaccharides [206]. TLR2, TNF-α, and IFN-γ are up-regulated in livers of rats in NASH [207].	BDE-209 enhanced TLR4-dependent lipid uptake in vitro in human macrophages [192].

## Data Availability

Not applicable.

## Abbreviations

ACC	acetyl-CoA carboxylase
AhR	aryl hydrocarbon receptor
ALT	alanine aminotransferase
AOP	adverse outcome pathways
AST	aspartate Aminotransferase
ATP	adenosine triphosphate
Bcl2	b-cell lymphoma 2
BFR	brominated flame retardants
cAMP	cyclic adenosine monophosphate
CAR	chimeric antigen receptor
CCR5	c-c chemokine receptor type 5
CD36	cluster of differentiation 36
cGAMP	cyclic guanosine monophosphate-adenosine monophosphate
cGAS	cyclic guanosine monophosphate-adenosine monophosphate synthase
ChREBP	carbohydrate-responsive element-binding protein
CYP450	cytochrome P450
DAMPs	damage-associated molecular patterns
DGAT	diacylglycerol acyltransferase
ERK	extracellular-signal-regulated kinase
FAS	fatty acid synthase
FR	flame retardants
FXR	farnesoid X receptor
GLUT4	glucose transporter type 4
HCC	hepatocellular carcinoma
HMGB1	high mobility group box protein 1
HFD	high-fat diet
IFN	interferon
IFNGR	interferon-gamma receptor
IL	interleukin
IRF	interferon regulatory factor
IRS	insulin receptor substrate
ISGs	interferon-stimulated genes
ISRE	interferon-stimulated response elements
JAK1	Janus kinase 1
JNK	c-jun N-terminal protein kinase
LXR	liver X receptor
MAPK	mitogen-activated protein kinase
MCP	monocyte chemoattractant protein
MDC	metabolic disrupting chemicals
MIE	molecular initiating events
mtDNA	mitochondrial DNA
mTOR	mammalian target of rapamycin
NAFLD	nonalcoholic fatty liver disease
NASH	nonalcoholic steatohepatitis
NLRs	nucleotide-binding oligomerization domain-like receptors
NR	nuclear receptor
NXR	nuclear xenobiotic receptors
OPFRs	organophosphorus flame retardants
PAMP	pathogen-associated molecular patterns
PBDEs	polybrominated diphenyl ethers
PBMC	peripheral blood mononuclear cells
PI3K	phosphoinositide 3-kinase
PPAR	peroxisome proliferator-activated receptor
PRR	pattern recognition receptors
PXR	pregnane X receptor
RAGE	receptor for advanced glycation end products
ROS	reactive oxygen species
RXR	retinoid X receptor
SCD-1	stearoyl-CoA desaturase-1
SOCS	suppressor of cytokine signaling
SREBP	sterol regulatory element-binding protein
STAT	signal transducer and activator of transcription
STING	stimulator of interferon genes
T2D	type 2 diabetes mellitus
TAFLD	toxicant-associated fatty liver disease
TASH	toxicant-associated steatohepatitis
TGFβ	transforming growth factor-beta
TH	thyroid hormone
TLR	Toll-like receptor
TNF	tumor necrosis factor
TYK2	tyrosine kinase 2
VLDL	very-low-density lipoprotein
